# Reasons for first line ART modification over the years during the ART scale up in Uganda

**DOI:** 10.1186/s12981-019-0246-y

**Published:** 2019-10-09

**Authors:** B. Castelnuovo, F. Mubiru, I. Kalule, A. Kiragga

**Affiliations:** Infectious Diseases Institute, College of Health Sciences, Makerere University, Mulago Hospital Complex, P.O. Box: 22418, Kampala, Uganda

**Keywords:** Antiretroviral treatment modifications, Toxicity, Sub-Saharan Africa

## Abstract

**Background:**

During the initial scale up of ART in sub-Saharan Africa, prescribed regimens included drugs with high potential for toxicity (particularly stavudine). More recently a growing number of patients requires second line treatment due to treatment failure, especially following the expansion of viral load testing. We aim to determine the reasons and risk factors for modification of first line ART across the years.

**Methods:**

We included patients started on standard first line ART (2NRTI + 1 NNRTI) between 2005 and 2016 at the Infectious Diseases Institute, Kampala, Uganda. We described the reasons for treatment modification categorized in (1) toxicity (2) treatment failure (3) other reason (new TB treatment, new pregnancy). We used Cox proportional hazard to identify factors associated with treatment modification due to toxicity.

**Results:**

We included 14,261 patients; 9114 (63.9%), were female, the median age was 34 years (IQR: 29–40), 60.8% were in WHO stage 3 and 4. The median BMI and CD4 count were 21.9 (IQR: 19.6–24.8) and 188 cell/µL (IQR: 65–353) respectively; 27.5% were started on stavudine, 46% on zidovudine, and 26.5% on a tenofovir containing regimens. We observed 6248 ART modifications in 4868/14,261 patients (34.1%); 1615 were due to toxicity, 1077 to treatment failure, 1330 to contraindications, and 1860 patients following WHO recommendation of phasing out stavudine and substituting with another NRTI. Modification for drug toxicity declined rapidly after the phase out of stavudine (2008), while switches to second line regimes increased after the implementation of viral load monitoring (2015). Patients with normal BMI compared to underweight, (HR: 0.79, CI 0.69–0.91), with CD4 counts 200–350 cells/µL compared to < 200 cells/µL (HR: 0.81− CI 0.71–0.93), and started on zidovudine (HR: 0.51 CI 0.44–0.59) and tenofovir (HR: 0.16, CI 0.14–0.22) compared to stavudine were less likely to have ART modification due to toxicity. Older patients (HR: 1.14 per 5-year increase CI 1.11–1.18), those in WHO stage 3 and 4 (HR: 1.19, CI 1.06–1.34) were more likely to have ART modification due to toxicity.

**Conclusions:**

Toxicity as reason for drugs substitution decreased over time mirroring the phase out of stavudine, while viral load expansion identified more patients in need of second line treatment.

## Background

During the initial scale up of ART in sub-Saharan Africa (SSA), recommended ART regimens included cheaper drugs [1], particularly stavudine, which are proved to be effective for the treatment of HIV [2], but have high potential for toxicity, [3–5]. In Uganda from 2003 to 2008 the typical ART regimens included stavudine or less frequently zidovudine, plus lamivudine plus nevirapine or efavirenz [6]. In 2008 the Uganda Ministry of Health (MoH) recommended a systematic national drug substitution from stavudine to other nucleoside/nucleotide reverse transcriptase (NRTI) regardless of the presence of toxicities [7, 8], preceding the 2010 WHO recommendation of replacing stavudine with zidovudine or tenofovir [9]. Tenofovir is an nucleotide reverse transcriptase inhibitor which has shown a better safety profile than stavudine and zidovudine [2], and several observational studies demonstrated that modifications in the first 2–3 years on ART due to toxicities are less frequent in patients started on tenofovir [5, 8, 10–13]. Other relatively common reasons for ART modifications in SSA, less common in resource rich settings, are contraindications due to tuberculosis treatment or pregnancy. Specifically patients who are on nevirapine containing regimens and diagnosed with tuberculosis are switched to efavirenz to avoid drug–drug interactions, and up to year 2012 [14] women who became pregnant while on an efavirenz were switched to nevirapine due to the fear of teratogenicity.

Simultaneously, along with the scale up of ART, a growing number of patients on first line ART have required second line treatment using algorithms informed by potential acquired [15] or transmitted resistance [16]. In most settings in SSA, treatment failure has been identified using clinical and immunological criteria as indicated by the World Health Organization (WHO) guidelines [1, 17]. Starting in 2010 [9], due to the suboptimal sensitivity and specificity of these criteria [18–20], the WHO suggested that, where possible, prospective viral load (VL) monitoring should be used. In Uganda routine VL monitoring scale up was initiated in December 2014, and VL testing has been performed at the national testing hub located at the Central Public Health Laboratories of the Uganda MoH.

In a context with limited treatment options there is need to sustain patients on a potent first line regimen as long as possible, while ensuring minimal toxicity and continuous efficacy of the drugs. ART modifications in busy clinical setting in SSA often have organizational and staff implications since a “modification visit” may last longer, due to additional counseling and education, and requires more trained staff; monitoring rates of ART modification is also critical in order to ensure continuity of drug supply. At patient level, even a single drug substitution can potentially disrupt the dietary or sleeping routine, as well as worsen adherence due to change of the drug timing or misunderstanding of the new schedule.

With this work we aim to determine the reasons and frequency of modification of first line ART regimens in HIV infected adults in a large urban over the years during the ART scale up in Uganda. We also aimed to determine factors associated to modification of first line ART due to toxicity.

## Methods

### Setting

The study was conducted at the Infectious Diseases Institute (IDI), a center of excellence for HIV treatment and care [21], with over 35,000 adult patients ever registered, and over 8000 active patients.

Since IDI inception in 2004, ART has been initiated and prescribed following the contemporary WHO guidelines [1, 9, 17], including the recommended CD4 count threshold, and the first line regimens of choice. At the IDI clinic ART was monitored using bi-annual CD4 count, combined with a confirmatory ad hoc VL [22] for patients suspected of treatment failure treatment failure. From December 2014 annual viral load testing became available for all patients nationally. Therefore, patients with treatment failure were initially identified using immunologic criteria according to the WHO guidelines; only patients with a confirmatory viral load testing > 1000 were switched to second line; after the implementation of viral load monitoring patients with 2 consecutive viral measurements > 75 copies/ml are considered for switch to second line.

### Patients and follow up

This study was a retrospective analysis of all patients started at IDI on standard first line ART (2NRTI + 1 non-nucleoside/nucleotide reverse transcriptase (NNRTI)) since the program inception in 2005 up to the end of 2016 with the closure of the database at the end of 2017 In our program at each visit after ART start the provider (a doctor or a nurse, depending on the general health condition of the patient [23]) takes the medical history, vital signs and performs physical examination. ART regimen, adherence, toxicity and laboratory tests are also reviewed, and ART and other medications are prescribed.

The information obtained during the clinic visits is entered in real time by the health providers into the IDI electronic medical record system, the Integrated Clinic Enterprise Application (ICEA) [24]. ART regimens are pre-coded and entered by choosing the correct code from a drop down. To eliminate the omission of important steps, or inconsistencies, automated queries were created within the database, and many fields are mandatory, and must be filled-in before the record can be considered valid and saved. In the context of ART regimens, if a health provider enters a regimen code different from the one entered on the previous visit, this activates a mandatory field asking for the “reason for ART change”. The reasons for ART change are also categorized and pre-coded and appear in a drop down. This check prevents both omitting the reason for ART change, but also erroneously entering and prescribing a regimen different from the one the patient is taking, in case no change was planned.

### Data collection and analysis

The following data was extracted from the electronic medical records database: gender age, body mass index (BMI), WHO stage, ART start date, CD4 count at ART start, ART regimen categorized by zidovudine, stavudine, and tenofovir based, and reason for ART modification.

We described baseline characteristics stratified by gender and we used Chi square for categorical variables and Kruskal–Wallis tests to compare proportions and medians for non-normally distributed variables.

We described the reasons of any treatment change by year categorized in (1) toxicity (2) treatment failure (3) contraindication (new tuberculosis (TB) treatment, new pregnancy). Patients switched off stavudine without experiencing drug toxicity were not categorized as “switched due to toxicity” but as a separate group where ART was modified following MoH recommendations of national drug substitution. We calculated time to first drug substitution due to toxicity in patients initiated on ART before and after 2008 (year of national switch from stavudine). We also obtained the probability of time to first drug substitution stratified by gender and initial ART regimen (categorized as stavudine, zidovudine and tenofovir based regimens) using Kaplan–Meier survival methods and compared using log rank test.

To identify factors associated with treatment change due to toxicity we used Cox proportional hazard model which included the following covariates: sex, baseline CD4 count (categorized as < 200, 200–350, and > 350 cells µL), age in 5-year increases, WHO stage, baseline body mass index (BMI categorized as: underweight: < 18.5; normal: 18.5–25; overweight: > 25), and regimen at ART initiation (zidovudine versus stavudine versus tenofovir based). Variables with P valued < 0.2 in the bivariate models were considered for inclusion in the multivariate Cox regression model.

## Results

A total of 14,792 patients were started on ART during the study period (2005–2016); 501 patients were started on triple NRTI or blinded regimens as part of clinical trials; 14,261 were started on standard first line regimes and were included in the analysis. The majority, 9114 (63.9%), were female, the median age was 36 years (IQR: 30–42), and the median time on the first line was 2.8 years (IQR: 0.6–5.7).

Table 1 displays the characteristics at ART start of all patients and stratified by gender.

**Table 1:** Characteristics at ART start by gender patients initiated on standard first line antiretroviral therapy at the Infectious Diseases Institute Kampala, Uganda

Characteristics	Overall N = 14,261 (100%)	Female N = 9114 (63.9%)	Male N = 5147 (36.1%)	P value
Age in completed years, median (IQR)	36 (30–42)	34 (29–40)	38 (33–44)	< 0.001
WHO stage 3 and 4, n (%)	7749 (60.8)	4533 (55.8)	3216 (69.7%)	< 0.001
BMI, kg/m^2^, median, (IQR) n (%)^a^	21.9 (19.6–24.8)	22.7 (20.1–25.8)	20.8 (19–22.9)	< 0.001
Underweight (< 18.5)	1975 (14.7)	1037 (12.1)	938 (19.4)	< 0.001
Normal (18.5–25)	8231 (64.4)	4926 (57.5)	3305 (68.4)	
Overweight (> 25)	3196 (23.9)	2604 (30.4)	592 (12.24)	
CD4 count, cells/µL, median (IQR)^b^				< 0.001 < 0.001
n (%)	188 (65–353)	210 (80–380)	153 (47–300)	
< 200 cells/µL	7274 (52.2)	4317 (48.2)	2957 (59.3)	
200–350 cells/µL	3139 (22.5)	2094 (23.4)	1045 (21)	
> 350 cells/µL	3519 (25.3)	2537 (28.4)	982 (19.7)	
Period of ART start, n (%)				0.682
2005–2008	6879 (48.2)	4408 (48.4)	2471 (48.0)	
2009–2017	7382 (51.8)	4706 (51.6)	2676 (52.0)	
ART regimen n, (%)				0.007
Stavudine	3932 (27.5)	2565 (28.2)	1367 (25.6)	
Zidovudine	6555 (46.0)	4213 (46.2)	2342 (45.5)	
Tenofovir	3774 (26.5)	2336 (25.6)	1438 (27.9)	

*IQR* interquartile range, *BMI* body mass index, *ART* antiretroviral treatment

^a^Missing for 859 patients

^b^Missing for 144 patients

Generally, females were younger (median age 34 years (IQR: 29–40), versus 38 in males (IQR) 33–44, P value: < 0.001). As compared to females, male patients were sicker at presentation and ART start, with a higher proportion in WHO stage 3 and 4 (69.7% versus 55.8%, P value: < 0.001), had a lower BMI (median 20.8 (IQR: 19–22.9) versus 22.7 (IQR: 20.1–25.8, P value: < 0.001), were more likely to be underweight (19.4% versus 12.1, P value: < 0.001), had a lower CD4 count [153 cells/µL (IQR: 65–353) versus 210 cells/µL (IQR: 80–380)] and had a higher proportion with CD4 count < 200 cells/µL (59.3% versus 48.2%, P value: < 0.001).

A similar proportion (46%) of males and females were started on zidovudine based regimens; a higher proportion of women were started on stavudine as compared to males (28.2% versus 25.6%), while a higher proportion of males being started on tenofovir based regimens as compared to females (27.9 versus 25.6%) (P: 0.007).

In total we observed 6248 ART modifications in 4868/14,261 patients (34.1%), of which 1130 (23.2%) had ≥1 modification; 1615 modifications were due to toxicity, 1077 due to first line treatment failure, 1330 for contraindications, and 1860 patients had ART modified following MoH recommendations of substituting stavudine with another NRTI.

**Fig. 1:**
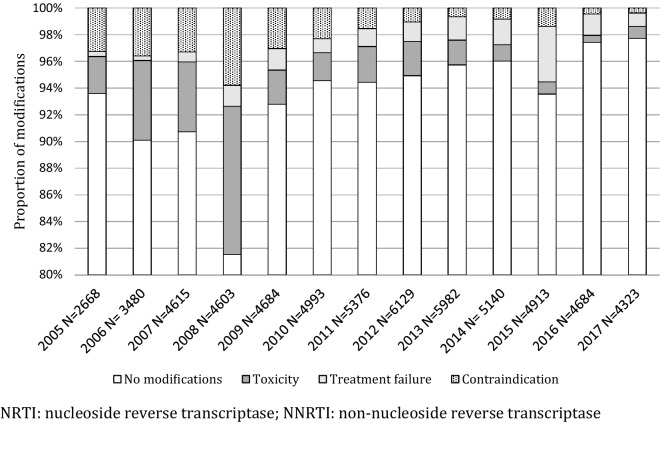
Reasons for antiretroviral treatment modification in patients started on standard antiretroviral first line regimen at the Infectious Diseases Institute

When comparing patients with single and multiple drug changes (for any reason) we found that in the group of patients with multiple switches there was a higher proportion of females (24.3% versus 21.2%, P = 0.17), of patients with CD4 count < 200 cells/µL (71.8% versus 62.9%), with advanced stage diseases (WHO 3 and 4 69.9% versus 64.8%, P = 0.002), patients started on stavudine (74.4% versus 56.5%, P < 0.001) and started in the 2005–2008 period (80.5% versus 69.0%, P < 0.001). Figure 1 shows proportion of patients with no ART modifications, modification for toxicity, treatment failure, and contraindication per year from 2005 to 2017. Of note the proportion of the total patients in care with ART modification for drug toxicity increased overtime in the first period from 2.7% in 2005 to 7.6% in 2008, declining rapidly to 2.4% in 2009, after the systematic phase out of stavudine, with only 0.8% at the end of the study period (2017). On the other hand, while an increasing proportion of patients per year were switched to second line due to treatment failure, the highest proportion (4.2%) was observed in 2015, during the first year of routine viral load monitoring. We also observed a declining trend in modifications for contraindication, from 3.1% in 2005 to 0.4% in 2017.

Overall the incidence of first drug modification for toxicity gradually reduced from 76.6 (CI 71, 6–81.8) per 1000 person years at risk in 2005 to 26.2 (CI 9.8–69.8) in 2017, with a drop from 38.9 (CI 36.7–41.2) in patients stated on ART before 2008 to 26.4 (CI 24.4–287) per 1000 person years in patients started from 2009 onwards (P < 0.001).

**Fig. 2:**
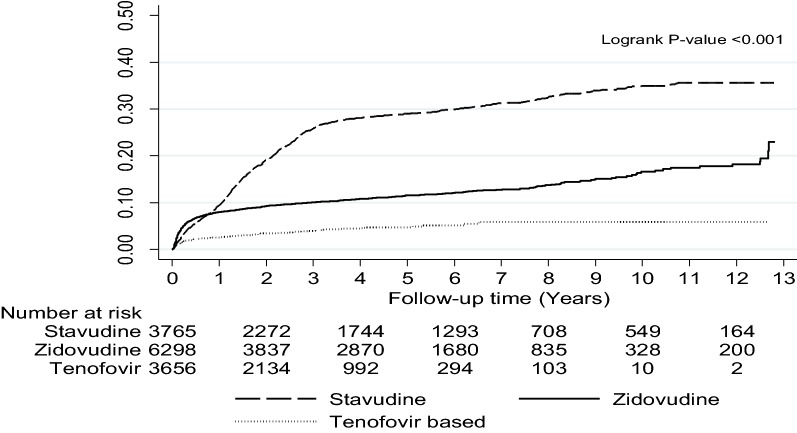
Cumulative probability of ART modification due to toxicity stratified by ART regimen in patients started on standard antiretroviral first line regimen at the Infectious Diseases Institute

In total 1733 (12.5%) patients had an ART modification due to treatment toxicity. We did not find a difference in the cumulative probability of ART modification due to toxicity by gender (males: 0.19, CI 0.17–0.21 versus females: 0.24, CI 0.22–0.26) (P = 0.15), while we observed a higher probability of ART modification in patients started on stavudine (0.36, CI 0.34–38) compared to patients started on zidovudine (0.19, CI 0.11–0.16) and tenofovir (0.06, CI 0.05–0.07) (P value < 0.001) (Fig. 2).

In multivariate analysis patients who were less likely to change drugs due to toxicity had normal BMI as compared to patients who were underweight, (HR: 0.73, CI 0.64–0.83, P value: < 0.001), had a CD4 counts between 200 and 350 cells/µL (HR: 0.73− CI 0.65–0.83, P value: < 0.001), as compared to < 200 cells/µL, and patients started on zidovudine (HR: 0.51 CI 0.44–0.59, P value: < 0.001) and tenofovir (HR: 0.16, CI 0.14–0.20, P value: < 0.001) as compared to stavudine. Older patients (HR: 1.14 per 5-year increase in age CI 1.11–1.18, P value: < 0.001), those in WHO stage 3 and 4 (HR: 1.19, CI 1.06–1.34, P value: < 0.001) were more likely to change regimen due to toxicity (Table 2).

**Table 2:** Cox regression analysis for risk factors for treatment modification due to toxicity in patients started on standard antiretroviral first line regimen at the Infectious Diseases Institute

Characteristics	HR	P	Adjusted AR	P
Gender				
Male	Ref	0.15	Ref	0.67
Female	1.08 (0.98–1.19)		1.03 (0.89–1.19)	
Age, per 5 years increase	1.13 (1.10–1.16)	< 0.001	1.14 (1.11–1.18)	< 0.001
WHO stage				
1 and 2	Ref		Ref	0.002
3 and 4	1.48 (1.33–1.64)	< 0001	1.19 (1.06–1.34)	
BMI kg/m^2^				
Underweight (< 18.5)	Ref		Ref	
Normal (18.5–25)	0.73 (0.64–0.83)	< 0001	0.79 (0.69–0.91)	0.001
Overweight (> 25)	0.71 (0.61–0.83)	< 0.001	1.92 (0.78–1.09)	0.334
CD4 count				
< 200 cells/μL	Ref		Ref	
200–350 cells/μL	0.73 (0.65–0.83)	< 0001	0.81 (0.71–0.93)	0.002
> 350 cells/μL	0.51 (0.45–0.58)	< 0.001	1.09 (0.89–1.34)	0.386
ART regimen (N, %)				
Stavudine	Ref		Ref	
Zidovudine	0.44 (0.39–0.48)	< 0001	0.51 (0.44–0.59)	< 0001
Tenofovir	0.16 (0.14–0.20)	< 0001	0.17 (0.14–0.22)	< 0001

## Discussion

In this work we describe the reasons for ART modification in a large urban clinic in Uganda over a long period (13 years) of time.

Most of the ART modifications were due to reasons other than treatment failure, consisting mainly in single drug substitution due to toxicity and contraindications. In our setting it is paramount minimizing any ART substitution, since once a drug combination is modified in absence of a viral load and a drug resistance test, ideally it should not be given to the same patient again. This in turn results in making the availability of future ART combinations rationed, in a context of an already limited variety of regimens. Additionally, if a modification occurs while a patient is unknowingly on treatment failure, it is likely that resistance to the new drug will develop. During most of the study period routine VL testing was not available; even after viral load testing was implemented, in our settings the use of viral load is restricted to ART monitoring at specific strict intervals (12 months).

Almost one quarter of the patients experienced more than a switch for any reasons; among patients with multiple switches we found a higher proportion of female, low CD4 count and advanced disease, ART start regimen containing stavudine and starter in 2005–2008,

Additionally, low CD4 count, and WHO stage 3 and 4, were found to more frequent in the group with multiple switches as compare to patients who experienced a single, suggesting that advance diseases and stavudine use may be associated to multiple events.

*Drug contraindications* as a reason for change were mainly attributed to NNRTI, particularly to nevirapine. Due to drug–drug interaction between nevirapine and rifampicin, these drugs are never co-administered in our settings. Of note, after a peak in 2008, drug modifications due to contraindications experience a decrease. We believe this was due to both a decrease in diagnosis of TB cases in our clinic [25], and the shift to using tenofovir, lamivudine/emtricitabine, and efavirenz as the preferred first line ART choice also in child bearing age women after the implementation of the 2012 WHO guidelines (EFV recommended as the NNRTI of choice in pregnant women).

*Toxicity* was a common reason for treatment modifications; overall tenofovir based regimens had a lower drug substitution as compared to stavudine and zidovudine, similarly to what is reported in studies from similar settings [5, 13]. Our study also shows that ART modifications due to toxicity reduced dramatically after stavudine phase out, demonstrating that the expected effect, the reduction in ART regimen modifications driven by side effects, was in fact achieved. Our study showed that sicker patients, reflected by low CD4 count, low weight, and more advance WHO stage of disease, are at risk of treatment modification due to toxicity.

Our results are consistent with previously published data that show that patients in more advance stage of disease are more likely to experience side effects [4, 26], probably due to new opportunistic infections, and to co-administration of other medications. This points out to another advantage of starting ART as early as possible, because in addition to other clinical benefits, it could increase the durability of first line ART.

In 2015 we observed a peak of ART modifications due to *treatment failure,* up to 4.2% of the patients in care. This sudden increase can be explained by the scale up of routine viral load monitoring in a setting were patients were earlier monitoring using only CD4 count; it is likely that a large number of patients who did not meet criteria for clinical and immunological failure, had a detectable viral load. It has to be noted that after 2015 we recorded lower rates of switch to second line (1.8% in 2016, 1% in 2017). This can be explained by several factors; first of all, in our specialized center we usually attain high levels of adherence to first line ART and viral suppression, even among patients on ART for a long time, with 95.8% of patients on first ART for 10 years having viral suppression [27]. Secondly, but not less important, delays in switching patients with viral failure have been reported from our [28] and other similar settings [29] and therefore this proportion presented may not reflect all patients in need of second line treatment. While viral load monitoring may have increased the detection of patients with treatment failure, it is important that these patients are timely switched to second line treatment.

Our study has some limitations. First of all, this is an analysis of a single treatment site, and additionally a specialized HIV care centre, and therefore only reflects practices at that centre, and may not be generalizable. Of note at our clinic we did not experience drugs stock outs or modification in ART regimen due to unavailability of other drugs as reported in our studies [30]. It has also to be noted that the low switches of patients on tenofovir may not reflect the magnitude of tenofovir related toxicity; annual creatinine for kidney toxicity has not been routinely performed because not supported by the national program, as well as bone mineral density measurements due to high cost of Dual-energy X-ray absorptiometry (DEXA). Another limitation is the possibility of informative censoring of patients lost to follow up, who may have disengaged from care due to drug side effects, as well as suboptimal adherence and treatment failure; we may have underestimated the actually magnitude of ART modifications required.

Additionally, before 2015 ART when ART was monitored using CD4 count which have low specificity to detect treatment failure [18, 31, 32], the numbers of patients in need to switch to second line treatment were certainly underestimated, similarly to other programs with no viral load monitoring [33]. For this reason, we did not perform a multivariate analysis to identify predictors of ART modification due to treatment failure. We also did not perform a similar analysis to assess factors associated to modification due to contraindication, since these reasons were heterogeneous. Finally risk factors for drug substitution for toxicity in the multivariate analysis take in account only the first drug substitution.

Despite being data routinely collected, an evaluation of the data in our database revealed low rate of missing and inconsistent data [24].

## Conclusions

In our study most of the ART modifications were due to reasons other than treatment failure, consisting mainly in single drug substitution due to toxicity and contraindications. The contribution of toxicity as reason for drugs substitution decreased over time mirroring the phase out of stavudine and the increasing availability of better tolerated regimes. Substitutions for contraindication also declined, due to a decrease in cases of tuberculosis and to the WHO recommendations of using efavirenz in pregnant mothers. New regimens including the integrase inhibitor dolutegravir are currently being expanded in some African countries including Uganda, calling for further future investigations on reasons for ART modification.

## Data Availability

The datasets used and/or analysed during the current study are available from the corresponding author on reasonable request.

## Abbreviations

ART: antiretroviral treatment
BMI: body max Index
DEXA: dual-energy X-ray absorptiometry
ICEA: Integrated Clinic Enterprise Application
IDI: Infectious Diseases Institute
MoH: Ministry of Health
NRTI: nucleoside reverse transcriptase
NNRTI: non-nucleoside reverse transcriptase
TB: tuberculosis
WHO: World Health Organization
